# Short-Time Changes of Intraocular Pressure and Biomechanics of the Anterior Segment of the Eye during Water Drinking Test in Patients with XEN GelStent

**DOI:** 10.3390/jcm11010175

**Published:** 2021-12-29

**Authors:** Joanna Przeździecka-Dołyk, Ewa Wałek, Agnieszka Jóźwik, Iwona Helemejko, Magdalena Asejczyk-Widlicka, Marta Misiuk-Hojło

**Affiliations:** 1 Department of Optics and Photonics, Wroclaw University of Science and Technology, 50-370 Wroclaw, Poland; agnieszka.jozwik@pwr.edu.pl (A.J.); magdalena.widlicka@pwr.edu.pl (M.A.-W.); 2 Department of Ophthalmology, Faculty of Medicine, Wroclaw Medical University, 50-367 Wroclaw, Poland; ewka.walek@gmail.com (E.W.); iwona.helemejko@gmail.com (I.H.); marta.misiuk-hojlo@umed.wroc.pl (M.M.-H.)

**Keywords:** intraocular pressure, primary open-angle glaucoma, minimally invasive glaucoma surgery, water-drinking test, corneal biomechanics, glaucoma

## Abstract

Purpose: Little is known about short-term changes in intraocular pressure (IOP) following minimally invasive glaucoma surgeries, such as post-XEN GelStent implantation. Although the importance of corneal biomechanics in glaucoma diagnostics has been reported, little work has been conducted on postoperative description of changes when the structure of the anterior segment is altered. The aim of presented study was to evaluate the changes in the biomechanical parameters of the anterior segment of the post-XEN GelStent implantation eyes. Patients and Methods: This investigator-initiated, open-label, prospective, single-center study recruited patients. Patients with primary open-angle glaucoma (POAG) after XEN GelStent implantation versus matched POAG controls (considered as control group/CG) treated pharmacologically were screened. Water loading was conducted using 10 mL of water per kilogram of body weight for ≤5 min. Goldmann applanation tonometry (GAT), corneal hysteresis (CH), and corneal resistance factor (CRF) were performed before water loading and after every 15 min up to 1 h. Results: The water drinking test (WDT) was positive in 3.7% (1 out of 27) of patients in the post-XEN group compared with 22.7% (5 out of 22) of patients in the control group (CG; *p* < 0.05). Mean fluctuations in GAT during the WDT were higher in the CG group (3.6 ± 2.5 mmHg vs. 2.9 ± 1.3 mmHg, *p* < 0.001). CRF and CH changed significantly only in the post-XEN group. The mean peak of CH and CRF occurred at 15 and 30 min of the test in the post-XEN group (*p* = 0.001). Conclusion: WDT is important to assess the ability of compensation mechanisms to reduce fluctuations in IOP after water upload. The relationship between biomechanics of the anterior segment and glaucoma may have substantial impact on surgical outcome evaluation.

## 1. Introduction

The water drinking test (WDT) was first described as a provocation test for glaucoma in the 1960s. It is a type of stress test which helps to assess the changes in eye pressure that may happen during the day. The WDT can be performed with a fixed volume of water upload or it can be adjusted for each person as 10 mL/kg body weight [1,2,3,4,5]. According to the review published by Susanna et al. (2017), an IOP peak in WDT that exceeds 5 mmHg is associated with six times increased risk of new onset of glaucoma. Interestingly, in the study reported by Poon et al. [4], WDT was considered as positive if IOP peak exceeded 6 mmHg, whereas Susanna et al. (2005) found that a significant difference of 1.9 mmHg in IOP peaks between the eyes that showed visual field progression and those that did not show any progress [2].

Interestingly, the WDT is experiencing renewed interest as the test that can indicate the impact of short-term changes in IOP after glaucoma surgeries.

In recent years, innovations in the field of glaucoma surgery have introduced minimally invasive glaucoma surgery (MIGS) devices that changed the therapeutic perspective. XEN GelStent (Allergan, Dublin, Ireland), one of the MIGS devices, is a 6 mm long stent of collagen-derived gelatin cross-linked with glutaraldehyde. Similar to trabeculectomy, it allows aqueous humor outflow from the anterior chamber into the subconjunctival space. It is placed ab interno with less damage to the ocular surface than the traditional methods. Moreover, its inner lumen diameter of 45 μm helps to limit the postoperative hypotony. The procedure is almost always augmented with subconjunctival or sub-Tenon injection of mitomycin C. The XEN GelStent received CE mark approval in 2011 and US FDA approval in 2016.

Accurate and clinically accepted standards for reproducible measurements of intraocular pressure (IOP) are crucial for proper diagnosis and treatment of glaucoma. Additionally, it is worth mentioning that IOP is not measured directly; rather, it is measured indirectly using contact methods and mathematical equations. Recently, several studies have described various factors, such as corneal curvature, central corneal thickness (CCT), and corneal biomechanical factors that significantly influence the IOP measurement [6,7]. Goldmann applanation tonometry (GAT) is accepted as the gold standard for intraocular measurements. However, GAT, similar to other methods of IOP measurements, is affected by CCT and corneal rigidity. In the era of minimizing the invasiveness of glaucoma surgery, the relationship between IOP and biomechanical parameters of the corneal or the anterior eye plays an increasingly crucial role in ophthalmology diagnostics [6,7].

A new generation of tonometers, such as Ocular Response Analyzer (ORA, Reichert Technologies, Depew, NY, USA) and Corneal Visualization Scheimpflug Technology (Corvis ST, Oculus, Wetzlar, Germany) allows to determine a biomechanical-independent IOP and to evaluate some biomechanical properties of the anterior eye and their correlations with eye pathologies. The ORA is a noninvasive air-puff tonometer that measures the biomechanical response of the eye to the impulse of air and some biomechanical properties of the cornea. Apart from the results related to IOP, such as Goldmann-correlated intraocular pressure (IOP_G_) and corneal-compensated intraocular pressure (IOP_CC_), the ORA generates two values related to corneal biomechanics: corneal hysteresis (CH) and corneal resistance factor (CRF). CH and CRF can demonstrate specific properties of the anterior segment in case of keratoconus, glaucoma, or diabetes [7,8,9]. It has been shown that IOP_G_ and IOP_CC_ are higher in the case of glaucoma when CH and CRF are lower [7,8,10,11]. Values of biomechanical parameters, such as CH and CRF, for glaucoma patients were found to be lower than those in the control group (CG) [7,8,10,11]. On the contrary, no association was found between anti-glaucoma medication and biomechanical parameters in the glaucomatous group [12]. In several studies, the association between lower values of CH and optic nerve and damage to the visual field in glaucoma patients has been reported [12,13]. Additionally, the risk of structural and functional glaucoma progression was shown to increase with the decrease in CH [13].

The present study investigated the impact of WDT on short-term changes in IOP in patients with XEN GelStent as compared to the primary open-angle glaucoma patients (POAG) treated with topical pharmacological eyedrops according to the European Glaucoma Society guidelines (EGS guidelines). The aim of the study was to evaluate the changes in the biomechanical parameters of the anterior segment of the eye caused by XEN GelStent implantation in the two analyzed groups.

## 2. Materials and Methods

The present study was an investigator-initiated, single-center, prospective, real-world evidence study that was registered in ClinicalTrials.gov (accessed on 5 April 2019) with the number NCT03904381 and conducted at the Department of Ophthalmology, Wroclaw Medical University. The clinical data base was collected between April and May 2018. Screening phase lasted between June 2018 and August and was conducted between September and December 2018. Informed consent was obtained from all subjects before the screening phase. The aims of the study, benefits, and risks of all procedures were thoroughly explained to the patients and the study was performed in adherence to the Declaration of Helsinki and was approved by the local ethics committee (approval number: KB 563/2017). The study included 39 patients in the screening phase after XEN GelStent implantation and 51 patients with POAG treated pharmacologically (CG) (for full details concerning number of patients involved in the process including screening, enrollment, and observation period see Table S1 in Supplementary Materials).

### 2.1. Patient Selection

Patients with POAG after at least 3 months and no more than 6 months of post-XEN GelStent implantation (IH) were prospectively enrolled in the study between September and December 2018 (EW, JPD). Unfortunately, there is little to no information about the time-dependent function of the implant, and in this area more research is needed. Technical details of implantation and perioperative period have been described previously [14]. Detailed inclusion and exclusion criteria for post-XEN implantation enrollment to WDT are shown in Table 1 and Table S2. Against this preselected group (post-XEN group), the matched (in terms of age, sex, refractive error, axial length, glaucoma progression, and retinal nerve fiber layer thickness) control of POAG (control group/CG) on local medications were enrolled (Table 1). None of the included subjects took any systemic anti-glaucomatous medication at least 3 months previously. In the post-XEN group, only three eyes (11%) required topical anti-glaucoma medications to control IOP (one patient was taking β-blocker and two patients prostaglandins eye drops). On the contrary, in the CG, all patients were on topical medication and 17 eyes (77%) required combined therapy of at least two drugs to control IOP. Patients from the control POAG group (CG) were taking β-blocker eye drops (19 eyes; 86%), prostaglandins eye drops (11 eyes; 50%), carbonic anhydrase inhibitor eye drops (6 eyes; 27%), and α-agonists (4 eyes; 18%).

### 2.2. General Ophthalmological Work-Up in Glaucoma Service

#### 2.2.1. Visit Regarded as the Standard Visit According to the Pre-Specified Protocol

Standard visits in the glaucoma service had been scheduled for all of the participants according to the study protocol (NCT03904381), the follow-up intervals as well as scheduled examination did not vary from the original version that was published in the clinical.trial.gov protocol and the conduction of WDT was considered as the additional procedure to assess the biodynamical response of the post-XEN GelStent after the water upload. All participants underwent complete ophthalmological examination before participation to determine their refractive and health status (JPD and EW). In the screening visit for both study groups, the visual field using standard white on white visual field (Humphrey Field Analyzer (HFA) II 750; 24-2 Swedish interactive threshold algorithm; Carl Zeiss Meditec, Dublin, CA, USA) and retinal nerve fiber layer thickness (RNFLT) using Spectralis OCT (Heidelberg Engineering, Heidelberg, Germany) were performed (JPD and EW). To unify the group, participants with cylindrical refraction ≥ 1.5 D were excluded from the analysis. On the day WDT was scheduled, the subjects’ refraction and keratometry (Speedy-K, Righton, Right Mfg. Co. Ltd, NIKON, Japanese.) and central corneal thickness (CCT) (PacScan300p; Sonomed Escalon, New Hyde Park, NY, USA) were measured.

#### 2.2.2. Additional Visit Related to the Water Drinking Test Procedure

During the WDT visit, the axial length (AXL), anterior chamber depth (ACD), and central corneal radius were measured with Lenstar (LE 900) prior to the water upload along with examination of endothelial cells (specular microscope): endothelial cells count (ECC) and their hexagenity; baseline readings of Goldmann applanation tonometry (GAT); and baseline ORA (Reichert Ophthalmic Instruments, Inc., Buffalo, NY, USA; Software version 3.0) (JPD, EW, and AJ), see example in Supplementary Figure S1. The specific set of examination was chosen due to the possible influence of corneal hysteresis (for full information regarding this aspect see Description S1 in Supplementary Materials). The biomechanical properties were measured thrice with waveform score > 5 each, and mean values were recorded for further analysis. During biometry recordings, five readings were automatically acquired and the mean was recorded. All recordings were conducted on the same equipment by the same dedicated examiner.

After the conduction of WDT, each patient was observed for a year and control visits were conducted as described in the study protocol (NCT03904381). No change in the original protocol follow-up visits or scheduled examination was made.

### 2.3. Water Drinking Test Procedure

**Figure 1:**
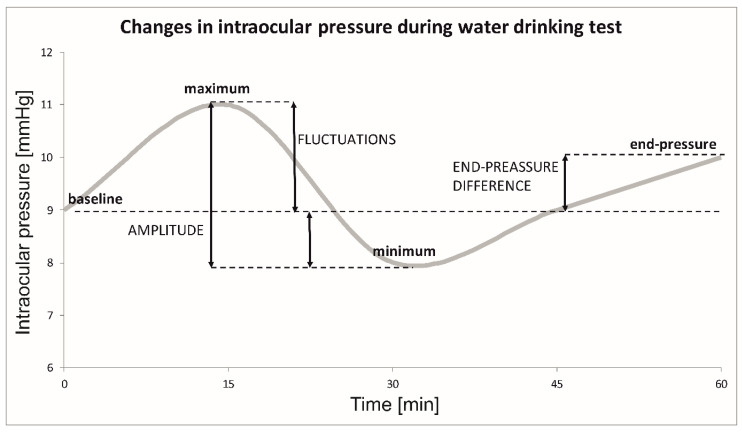
Changes in the intraocular pressure during the water drinking test.

Globally acceptable WDT was performed between 1 pm and 4 pm owing to a known diurnal variation of IOP [3,4]. The participants were asked not to ingest any food or liquid 4 h before the test. After recording baseline measurements and weighing each participant, all subjects were asked to drink 10 mL of water per kilogram of body weight for not more than 5 min. The measurements of ORA and GAT were obtained at the baseline and at 15, 30, 45, and 60 min (termed as end-pressure) after water loading (JPD, EW, and AJ). The peak IOP was defined as the maximum IOP measured within 1 h after water intake. The GAT and ORA recordings were evaluated separately. The maximum IOP fluctuation was defined as the difference between the peak IOP and baseline IOP measurements during the test. Additionally, the amplitude of IOP was recorded as the difference between the lowest and the highest IOP measurements during WDT (including baseline IOP) (Figure 1). Each test was assessed as positive or negative according to the pre-defined criteria. We considered WDT as positive if fluctuations of IOP were >5 mmHg and negative if these were ≤5 mmHg. We also assessed the differences in the categorized peak of IOP measured by GAT (peak of GAT) between both groups. The categories were as follows: >15 mm Hg, >18 mm Hg, >21 mm Hg, >24 mm Hg, >27 mm Hg, and >30 mm Hg.

### 2.4. Statistical Analysis

Statistical analyses were conducted using the MedCalc statistical software version 17.9.7 (MedCalc Software bvba, Ostend, Belgium; http://www.medcalc.org; accessed on 4 October 2017) and Statistica Software version 13.3 (TIBCO Statistica 1984–2017 TIBCO Software Inc., Palo Alto, CA, USA) licensed by University of Science and Technology in Wroclaw and Wroclaw Medical University, respectively. Sample size calculation was conducted assuming a pooled standard deviation of 2.4 units, the study would require a sample size of: 23 for each group (i.e., a total sample size of 46, assuming equal group sizes), to achieve a power of 80% and a level of significance of 5% (two sided), for detecting a true difference in means between the test and the reference group of −2 (i.e., 16.8–18.8) units. In other words, a selection of a random sample of 23 from each population, and determination that the means of the test and the reference groups are 16.8 and 18.8 units, respectively, and the standard deviation being 2.4 units, provide an 80% power to declare that the two groups have significantly different means, i.e., a two sided *p*-value of less than 0.05 [15]. The Shapiro–Wilk test was used to check the normality of the sample distribution. The F-test for equal variances was used to check the variance homogeneity. Intergroup comparisons were performed using Mann–Whitney *U* test for continuous variables and chi-square test for categorical variables. Comparisons of IOP before and after water intake in post-XEN and control groups were evaluated with Wilcoxon signed-rank test. Regression analysis was used to determine factors associated with IOP fluctuations during WDT. Repeated measures analysis of variance (ANOVA, Kruskal–Wallis, and Friedmann) with the Bonferroni adjustment for multiple comparisons was used to determine the influence of within-subjects factor water upload, and between-subjects factors (subject groups: post-XEN and POAG). Differences were considered statistically significant at *p* < 0.05. All data are expressed as mean ± standard deviations, 95% confidence interval (95% CI) for mean or median, and interquartile range (IQR) where applicable, depending on whether normal distribution was achieved or not.

## 3. Results

The study included the post-XEN group with 27 eyes, and age-, sex-, and IOP values-matched POAG control group (CG) consisting of 22 eyes. The median age for both groups was 68 years (IQR: 63–70 years and 65–72 years, respectively, for post-XEN and control groups; *p* > 0.05). Both the groups had similar female/male ratio (*p* > 0.05). No differences in the baseline GAT, IOP_CC_, IOP_G_, CH, and CRF, spherical equivalent, central corneal radius, CCT, ACD, AXL, BCVA, MD, PSD, RNFLT, body weight, BMI, ECC, and hexagonity of endothelial cells were recorded (Table 2).

### 3.1. Intraocular Pressure during the WDT

**Figure 2:**
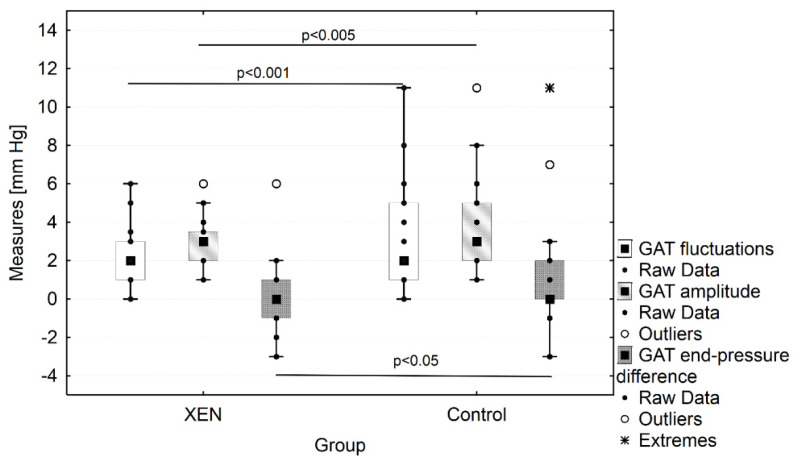
Differences in the WDT parameters such as GAT fluctuations, GAT amplitude, and GAT end-pressure difference between post-XEN and control group.

**Figure 3:**
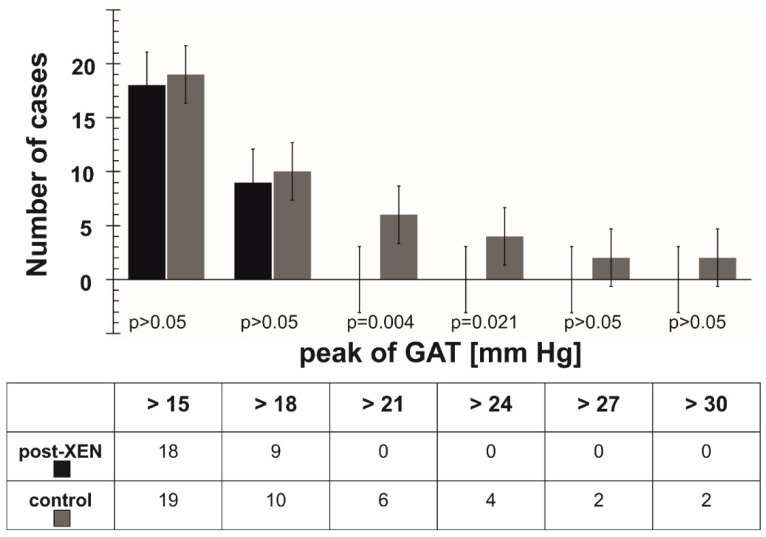
Comparison of the categorized GAT peak between the post-XEN and control groups (number of cases), Fisher’s exact test.

According to the pre-specified criteria, WDT was positive (the fluctuations of IOP were taken into consideration) for 3.7% (one patient) in the post-XEN group and 22.7% (five patients) in the CG (*p* = 0.045). Interestingly, when we considered the amplitude of IOP (fluctuations between any measurements during the test; difference between highest and lowest IOP value, including baseline IOP) during WDT ≥ 5 mmHg, significant and rapid changes were observed: 18.5% (five patients) and 31.8% (seven patients), respectively, in post-XEN and control groups (*p* = 0.84) (Figure 2). There was a significant change in the GAT peak and fluctuation times as well as values between the groups (Figure 2 and Figure 3). In the post-XEN group, there was an increase in the GAT between the 15th and 45th min (median Δ*GAT* = 1 mmHg), whereas the *peak of* GAT appeared at the 30th min in the CG (median Δ*GAT* = 2 mmHg; *p* = 0.031). Mean fluctuations in GAT during WDT were higher in the CG than in the post-XEN group (3.64 ± 2.54 mmHg vs. 2.91 ± 1.32 mmHg; *p* < 0.001; Figure 2 and Figure S2 in Supplementary Materials). Time-dependent changes in all measured parameters are presented in Table 3.

### 3.2. Intraocular Pressure during WDT as a Prediction Factor

Positive WDT in the post-XEN patient revealed the cause of increased RNFL loss (−4.51 μm/year) during the 12 month observation period compared to the mean value of all post-XEN group of −0.68 μm/year. Additionally, in all five post-XEN patients with positive WDT (according to the amplitude criterion) the increased loss of RNFL from the mean value of the group was observed (*p* = 0.048). Moreover, the magnitude of GAT increase 15 min after the test started was positively correlated with RNLF loss (*r* = 0.43, *p* = 0.024) during the 12 month period. There were also significant changes in categorized peak of GAT (in category >21 mm Hg and >24 mm Hg) between both groups (Figure 3 and Figure S2 in Supplementary Materials).

Further analysis reveal that the magnitude of GAT increase in 15 min was the best parameter in stepwise regression model constructed to predict RNFL loss (*β* = 0.68, *p* = 0.018). In our study, the most important outcome was the significance of the WDT in the post-surgical procedure outpatient care. As indicated in the Table 3, as well as Figure 2, Figures S2 and S3 in Supplementary Materials, when we performed the analysis with Mann–Whitney *U* test (comparing the two dependent samples—post-XEN and CG only as time points; each measurement separately) there was no significant difference in contrast to the analysis with the Friedman ANOVA test (comparing these same samples as the time-dependent parameters of two groups—as per repeated measurements in the two different groups). Both analyses show a different approach: for one (Mann-Whitney *U* test, Table 3), it indicates that we cannot differentiate both groups on the basis of a single measurement or even make the important conclusion about WDT due to the lack of significance between groups; for the second (Friedmann ANOVA test, Figure 2, Figures S2 and S3 in Supplementary Materials), only the full analysis of whole WDT results gives more information that the sum of the single result e.g., if the patient came to the office and during the visit had several GAT measurements irrespective to the water upload and time of the repeated measures, this will not give us any additional information about the dynamic response (Table 3). In contrast, if this same patient underwent WDT during the visit, the dynamic response information can be obtained and as in this research it can indicate a correlation with the RNFL loss in future (Figure 2, Figures S2 and S3 in Supplementary Materials). The presented analysis has a great impact on every-day practice, as we wish to observe more closely the patient that is positive to the WDT after surgery due to the fact that he is a possible candidate for rapid decreased RNFL.

### 3.3. Biomechanical Changes during the WDT

**Figure 4:**
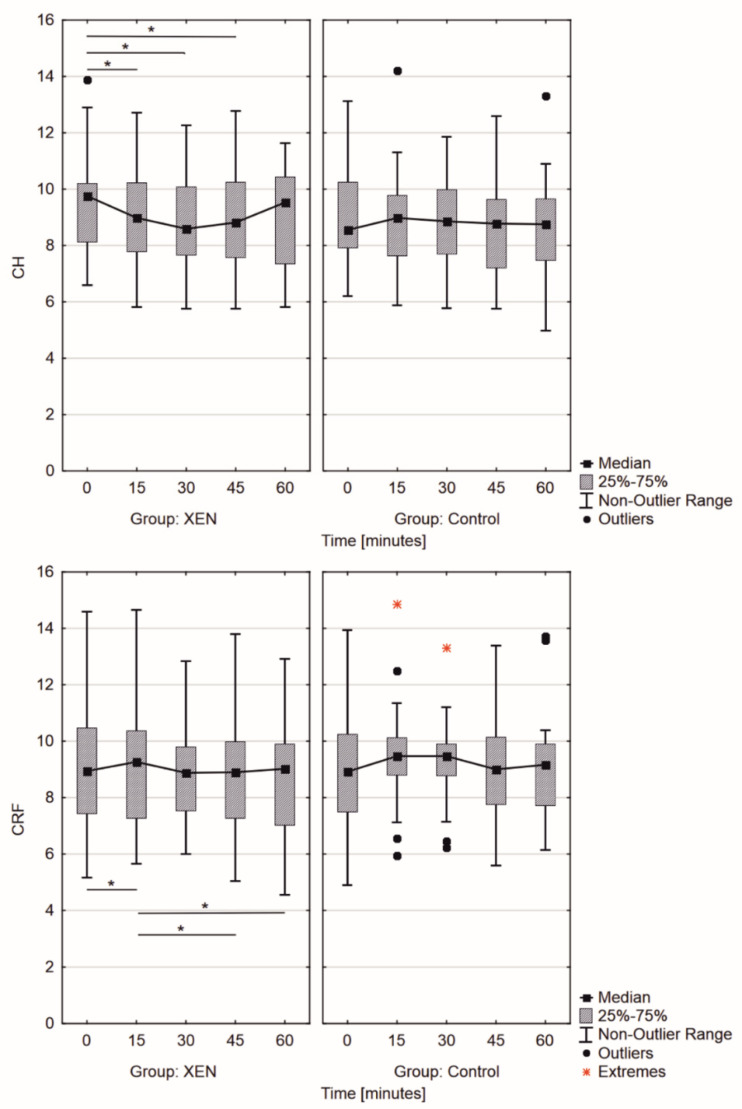
Changes in (**top**) corneal hysteresis (CH) and (**bottom**) corneal resistant factor (CRF) measured by Ocular Response Analyzer in the examined groups during the water drinking test. (* *p* < 0.05, Friedmann test).

**Figure 5:**
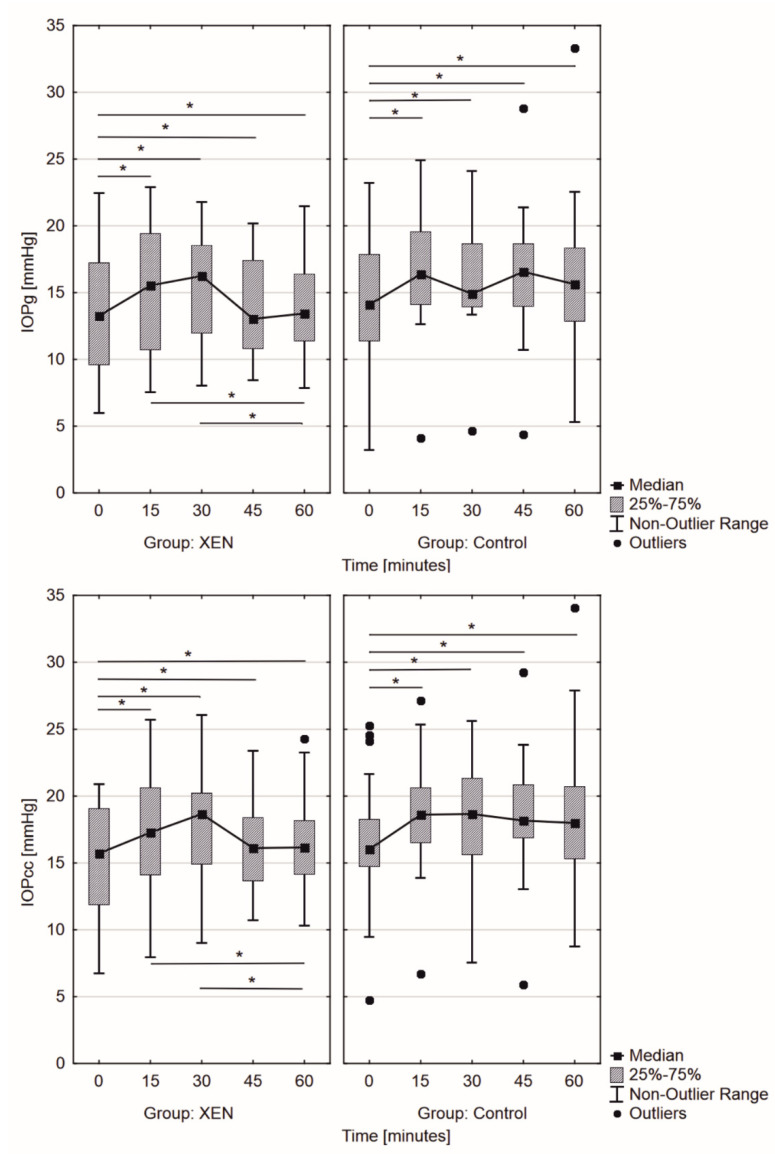
Changes in (**top**) Goldman-compensated intraocular pressure measured (IOP_G_) and (**bottom**) corneal-compensated intraocular pressure (IOP_CC_) measured by Ocular Response Analyzer during the water drinking test in the examined groups. (* *p* < 0.05, Friedmann test).

The biomechanical parameters of the anterior chamber in the post-XEN group changed significantly during WDT (*p* = 0.001 for both CRF and CH), whereas the same parameters in the CG presented no statistical changes in WDT (*p* = 0.66 and *p* = 0.08, respectively, for CRF and CH). The CH change was greater during WDT in the post-XEN group than in the CG (*p* = 0.02; Figure 4). Additionally, the peak of CH was observed at 30 min (median Δ*CH* = 1.15) after the start of the test in the post-XEN group (trend of CH changes with rank coefficient *r* = 0.067). In the CG, there were no statistically significant changes in CH, the variation in CH was observed in the first 15 min (median Δ*CH* = 0.43) from the start of the test. Moreover, this biomechanical parameter remained stable after that. Interestingly, the greatest change in CRF was observed in the post-XEN group within the first 15 min (median Δ*CRF* = 0.33; *r* = 0.067, *p* = 0.03), following which the CRF decreased to pre-WDT values. In the CG, it remained statistically insignificant with the peak revealing itself between the 15th and 30th min (median Δ*CRF* = 0.55, *p* = 0.66; Figure 4). In the post-XEN group, we observed significant increase (*p* < 0.001) in IOP_G_, with its peak after 30 min (median Δ*IOP_G_* = 2.99 mmHg), whereas two peaks at the 15th and 45th min were observed in the CG (median Δ*IOP_G_* = 2.46 mmHg; Figure 5). Significant differences were observed in IOP_CC_ measurements (*p* < 0.001); IOP_CC_ showed a gradual increase (median Δ*IOP_CC_* = 2.96 mmHg) in the post-XEN group over first 30 min with a decrease afterward, whereas the increase was observed only within the first 15 min of the test in the CG (median Δ*IOP_CC_* = 2.55 mmHg) and remained stable afterward, although at elevated levels (Figure 5).

## 4. Discussion

Our study has shown that the WDT test is useful in daily practice because this can be an indicator of rapid RNFL decrease. The present study compared the fluctuations in GAT and ORA measurements in post-XEN patients during the WDT, with those in the matched control group patients on local anti-glaucoma medications. The impact of systemic disease, such as diabetes, on anterior biomechanical parameters can be confounding.

In recent years, the WDT is once again in the focus of attention of some scientists. Research that evaluates the IOP peak (highest IOP after drinking water), fluctuations (difference between IOP peak and baseline), amplitude (difference between highest and lowest IOP value, including baseline IOP), or end-pressure difference (IOP at 60 min versus baseline) differs in reported values. It is worth mentioning that little is known about the amplitude during WDT, as most researchers focus their findings only on fluctuations, end-pressure difference, and in some cases range IOP (difference between IOP peak and IOP trough after drinking water, excluding baseline IOP) or IOP trough (the lowest IOP after drinking water) [6].

The post-trabeculectomy characteristics of IOP fluctuations, range, and end-pressure difference were respectively: 1.3–6.0 mm Hg, 2.1–4.9 mm Hg, and 0.1–3.2 mm Hg [6,7,8]. The similar characteristics of post-tube, post-SLT, and pharmacologically treated open-angle glaucoma group were respectively: (1) IOP fluctuations 3.6–6.8 mm Hg, 2.6 mm Hg, and 3.8–7.1 mm Hg, (2) IOP range/amplitude 2.8–4.6 mm Hg, 0.8 mm Hg, and 1.5–5.6 mm Hg, (3) end-pressure difference 5.6 mm Hg, 1.5 mm Hg, and 1.1–3.4 mm Hg [4,6,7,8,9,10].

Selection of control group was dictated by the well-described changes in intraocular pressure as well as anterior chamber biomechanics during the water drinking test in the medically treated glaucoma patients. The first choice control group appears to be post-trabeculectomy glaucoma patients, but after an intense literature search and clinical specialists advise we found that in this group there are too many differences in the perisurgical procedures as well as in post-surgical care, that it would not be possible to obtain a homogenic group. The additional procedures conducted in the post-trabelculectomy group were: self-massage of the blep conducted by the patient, change of pressure induced by the adjustable sutures, suturolisis, needling, 5-fluorouracil (5-FU) injection, mitomicin injection, and revision of trabeculectomy. On the contrary, the post-XEN group was a homogenic group of patients with only one having a post-surgical additional procedure conducted (5-FU injection).

Little is known about the results of WDT in post-tube surgery patients. Most of the publications that describe the WDT results focus on the pharmacological treatment results and on surgical intervention (i.e., trabeculectomy or tube implantation). On the other hand, we can observe in daily practice the tendency towards MIGS. The surgeons as well as day-to-day practice tendencies are shifting towards early minimally invasive glaucoma surgery. In this circumstance, the new question arises: “Do we know how the stent, tube, or other MIGS will behave during the stressed situation such as a water upload?” and another one “Is the patient equally protected during this stressed situation, as in case of pharmacological treatment after trabeculectomy?”. The questions are even more than we are able to answer. To the best of our knowledge, the present study is the first of its kind to study post-XEN GAT changes during WDT on the basis of biomechanics of the anterior chamber of the eye.

Previously, Razeghinejad et al. (2017) described the differences in WDT results in post-trabeculectomy and post-tube surgery [16]. According to his work, the GAT increased significantly from baseline in both the groups (the post-trabeculectomy as well as post-tube group; *p* < 0.001). In the post-trabeculectomy group, the average GAT increased significantly at 30 min; however, it decreased to some extend at 60 min, but not to the baseline GAT. In the post-tube group, GAT increased incrementally until the last measurement [16], see also the summary of results in Table 4. Unfortunately, the authors did not recognize the amplitude from fluctuation during GAT. Probably in the presented groups, both parameters were similar. Presented results stand in contrast from our studies, as we found that the GAT in post-XEN GelStent behaved in the flow-dependent way—early stage increase in GAT decreased nearly to the baseline values and in some patients we could observe even lower GAT values. The negative end-pressure difference was observed in eight patients (29.6%) from the post-XEN GelStent group. Similar results to those discussed previously were presented by Martinez et al. (2017), who reported that the results of WDT in post-trabeculectomy versus post-tube patients did not differ significantly (*p* = 0.854). Baseline GAT for post-trabeculectomy and post-tube groups were comparable, as well as the peak GAT values (*p* = 0.954) [17]. In the presented study, we do not have information about the end-pressure difference, on the other hand the range values of GAT were presented, see Table 4. In comparison to the above reports, the GAT values obtained in the present study indicated a smaller maximum increase after 30 min in the post-XEN group (Table 3 and Table 4).

In contrast to studies described above, Danesh-Meyer et al. (2008) reported lower WDT results for the post-trabeculectomy group than those for the pharmacologically treated group, see Table 4 [18]. However, considering all different surgery strategies, the XEN GelStent appeared to be as potent as classical trabeculectomy in preventing diurnal GAT peak; however, a “head-to-head” treatment comparison and WDT results are required to be presented.

Razeghinejad et al. (2017) reported that the end-pressure difference (intraocular pressure/GAT at 60 min vs. baseline) was significantly higher in the post-tube group than in the post-trabeculectomy group (*p* = 0.03) [16]. In the present study, the end-pressure in the post-XEN group did not differ significantly from the baseline, whereas the CG reported an increase in the end-pressure (*p* < 0.05). Martinez et al. (2017) described insignificant fluctuations in GAT (*p* = 0.618) and IOP amplitudes (*p* = 0.959) [17]. In contrast to this finding, Danesh-Meyer et al. (2008) described GAT statistically significant fluctuations between the surgically and pharmacological treated patients (*p* < 0.001), see Table 4 [18]. In our study, the fluctuations were closer to those in post-trabeculectomy groups as presented by Martinez et al. (2008) [7]. Nevertheless, our study is the first attempt to describe the post-XEN diurnal fluctuations in GAT and its origins. Moreover, a head-to-head comparison between post-XEN and post-trabeculectomy groups is required.

RNFL physiological slop differs between studies from −0.44 to −0.54 μm/year on the other hand, and RNFL slop in glaucomatous patients without progression range from −0.98 to −1.23 μm/year [21,22,23,24,25]. Compared to the literature, the presented mean RNFL loss of −0.68 μm/year is a very good result. Lin et al. described that the progressive RNFL thinning was confirmed by trend-based progression analysis, the mean and the peak rates of RNFL thinning were 9.06 µm/year and 4.52 µm/year, respectively [26]. When we consider this, our peak RNFL loss of −4.51 μm/year during a 1 year observation period in the patient that was WDT positive appeared to bare significant information for clinicians. Close observation of patients during the WDT may be helpful to identify patients that needs to be followed-up closely, regardless of IOP measurements. Our study shows that positive WDT based on the classical peak GAT and on the fluctuations GAT criteria enables us to find patients that are approaching the progression, while other parameters are insignificant. It is worth underlining that the magnitude of GAT increasing at 15 min was positively correlated with greater RNFL loss.

The Ocular Response Analyzer is a unique device that measures two intraocular pressures, namely IOP_G_ and IOP_CC_, and two parameters, namely CH and CRF, based on the biomechanical properties of the cornea, such as viscoelasticity. Literature reports no normative values for parameters, such as CH, CRF, IOP_G_, and IOP_CC_. The published ranges of these parameters in healthy study population vary slightly from study to study [11,13,14,15]. Values of biomechanical parameters, such as CH and CRF, for glaucoma patients were found to be lower than those in the control group (CG) [11,13,16,17,18]. On the contrary, no association was found between anti-glaucoma medication and biomechanical parameters in the glaucomatous group [19]. In several studies, the association between lower values of CH and optic nerve and damage to the visual field in glaucoma patients has been reported [17,19]. Additionally, the risk of structural and functional glaucoma progression was shown to increase with the decrease in CH [17].

The results obtained from ORA are valuable from the perspective of glaucoma diagnosis and selecting the therapy methods and predicting their effects, especially the results obtained for the post-XEN group. The interesting finding is that changes in CH and CRF during WDT (values as well as trend changes, Figure 4) showed statistically significant (Kruskal–Wallis test; *p* < 0.05) ability to distinguish the post-XEN group from control POAG patients (CG) (surgical intervention and pharmacological treatment, respectively). The control POAG group (CG) results of CH changes/Δ*CH* (from 8.8 ± 1.9 to 9.2 ± 1.9 mmHg) within the first 15 min were in agreement with those published by Ulas et al. (2014) (CH changes/Δ*CH* from 10.8 ± 1.4 to 10.5 ± 1.6 mmHg) regardless of absolute values. Changes in CH in the post-XEN group ranged from 9.4 ± 1.8 to 8.9 ± 1.7 mmHg within the first 15 min of WDT [27]. However, there is no literature on the changes in biomechanical properties during WDT for the post-XEN group. Based on the obtained results, we put forward the hypothesis that CH as well as CRF describes not only the corneal biomechanical properties but could probably also be considered a surrogate of biomechanical properties of the complete anterior segment. However, the uveal biomechanics cannot be derived from outflow alone, as this is only a small piece of the puzzle. More prospective investigations need to be conducted to fully understand this mechanism.

An interesting finding was that during WDT, the IOP_CC_ peak value for both groups did not differ significantly. However, it increased in the control POAG group (CG) during WDT in the first 15 min and remained stable afterward, in contrast to the post-XEN group, where it reduced right after the peak, followed by decreasing nearly to baseline values (different trends in both groups, *p* < 0.05; Figure 5). According to Ulas et al. (2014), IOP_CC_ significantly increased in the control POAG group (CG) from 15.3 ± 2.4 at baseline to 16.7 ± 2.8 mmHg at the 10th min (*p* < 0.001) [27]. The changes in the biomechanical properties of the anterior segment of the globe following XEN GelStent implantation segment could compensate for the fluctuations in GAT during WDT, and this mechanism protected the globe from the peak of GAT. This conclusion is driven from the observations suggesting that even if the baseline GAT was similar, in both groups in the post-XEN group there is an additional factor that stabilizes GAT during WDT, probably by means of biomechanical properties. The function of the implant itself to the WDT is the most probable cause. The most interesting finding was that the end-pressure in the post-XEN group was frequently lower than the baseline.

Postoperative assessment of the effectiveness of anti-glaucoma procedures (treatment) in reducing IOP based only on IOP measurements may be underestimated/overestimated, owing to the interference of the anterior segment with the structure, and thus changing the biomechanical parameters in this area. The results obtained in the WDT test showed changes in the biomechanical parameters in the matched (age, sex, AL, RNFLT, refractive error and glaucoma progression) group of patients after surgery compared to the pharmacological treatment. The results of the present study suggested that a study of the fluctuations in the biomechanical parameters during WDT could enable a more precise selection of the surgical procedure.

The statistical significance of CH and CRF parameters in glaucoma diagnosis has been reported previously [9,28,29,30]. However, most studies presented the comparison of ORA parameters between the healthy and glaucomatous eyes. Little work has been conducted on the postoperative diagnosis. Kotecha et al. (2007) reported the importance of CH and CRF parameters for the clinical diagnosis of corneal structure disorders such as Fuchs endothelial dystrophy, keratoconus, or refractive surgery. The findings of the present study indicated that analysis of ORA parameters could be utilized in postoperative and treated glaucoma diagnostics [31,32].

### Limitations of Our Study

The lack of WDT performed before surgery, as this is not a standard procedure during qualification for antiglaucoma surgeries and could possibly harm patients with increased intraocular pressure. This is a statistical limitation, but taking into account good clinical practice regulations and patient best care, it was unethical to conduct such test. We could include only patients in which the WDT can be safely performed before antiglaucoma surgery, but more importantly this was meant to present real-life data, and as such we could not exclude patients with high IOP and perform WDT only where it is safe. This kind of approach would result in the deviation of the study group and lose the most important clinical perspective—real-life data view.

The control group was pharmacological and not a surgical one. It could be better if a surgical control group was introduced e.g., trabeculectomy. On the other hand, there are great differences in conducting the trabeculectomy between different surgeons as well as patients (local conditions are different). The clinical trial comparing the biomechanical properties after XENGelStent and trabeculectomy needs to be thoughtfully planned and prepared (a multi-center study with large number of participants). Another issue is that for MIGS procedures, different patients are qualified than for trabeculectomy, as there is a difference in risks and IOP-lowering potential in those procedures. As such, MIGS can be more closely associated and compared with pharmacological treatment than classical surgeries.

In conclusion, the relationship between ocular biomechanics and glaucoma is still unknown; however, it offers potential for further investigations, the results of which may have a substantial impact on future glaucoma diagnosis as well as its treatment.

## Figures and Tables

**Table 1:** Inclusion and exclusion criteria for post-XEN and POAG groups.

	Inclusion Criteria	Exclusion Criteria
post-XEN group	At least 3 months and up to 6 months post XEN Gel stent implantation. At least 3 months post last 5-FU injection. Reduction of IOP compared to the pre-XEN implantation measurements of at least ≥20% baseline IOP and ≤21 mmHg.	Any progression (according to the EGS guidelines) within last 3 months. Any change in medication within last month. Any systemic medication within last 3 months. More than 3 5-FU injections. High axial refractive error due to elongation of the globe (AXL > 26 mm)
POAG group	At least 3 months on stable local anti-glaucoma medications without significant side effects. Reduction of IOP compared to the pre-medication measurements of at least ≥20% baseline IOP and ≤21 mmHg. Matched to the post-XEN group in terms of baseline GAT, IOP_CC_, IOP_G_, CH, and CRF, spherical equivalent, central corneal radius, CCT, ACD, AXL, BCVA, MD, PSD, RNFLT, body weight, BMI, ECC, and hexagonity of endothelial cells.	Any progression (according to the EGS guidelines) within last 3 months. Any procedures using laser. High axial refractive error due to elongation of the globe (AXL > 26 mm)

IOP—intraocular pressure, EGS—European Glaucoma Society, 5-FU injection—5 fluorouracil injection, GAT—Goldmann applanation tonometry, IOP_CC_—corneal-compensated intraocular pressure, IOP_G_—Goldmann-correlated, CH—corneal hysteresis, CRF—corneal resistance factor, CCT—central corneal thickness, ACD—anterior chamber depth, AXL—axial length of the globe, BCVA—best-corrected visual acuity, MD—mean deviation (perimetry), PSD—pattern standard deviation (perimetry), RNFLT—retinal nerve fiber layer thickness, BMI—body mass index, ECC—endothelium cells count.

**Table 2:** Demographic and clinical characteristics of the study groups (post-XEN and POAG).

	Post-XEN *N* = 27		POAG/Control *N* = 22		
	Mean ± SD	95% CI	Mean ± SD	95% CI	*p*-Value *
Age (years)	65 ± 10	61–69	68 ± 8	63–72	0.62
SE (D)	−0.50 ± 0.75	−2.25–1.50	−0.25 ± 0.75	−2.5–1.75	0.54
CCT (µm)	530 ± 36	516–544	535 ± 41	516–555	0.55
ACD (µm)	3.24 ± 0.77	2.91–3.57	3.05 ± 0.76	2.65–3.46	0.51
AXL (µm)	23.7 ± 1.7	23.1–24.4	23.6 ± 1.4	22.8–24.1	0.73
BCVA (logMAR)	0.06 ± 0.10	−0.02–0.22	0.08 ± 0.12	0.00–0.20	0.81
BMI (kg/m^2^)	21.6 ± 3.1	20.5–25.2	23.1 ± 2.8	21.6–25.1	0.62
MD (dB)	−8.8 ± 8.1	−11.1–−6.5	−8.7 ± 8.5	−13.0–−4.3	0.92
PSD (dB)	6.1 ± 4.1	4.9–7.2	6.1 ± 3.9	4.1–8.1	0.74
RNFLT (µm)	62 ± 11	-	64 ± 14	-	0.87
CD (count)	2049 ± 481	1859–2239	1818 ± 483	1598–2038	0.06
Hexagonity of endothelialcells (%)	54 ± 35	41–68	55 ± 29	41–68	0.94
Time ^†^ (months)	9 ± 2	7–11	10 ± 2	8–12	0.75

* Mann–Whitney *U* test; ^†^ time after surgery (post-XEN group) or after introduction of the treatment (control group). CCT—central corneal thickness, ACD—anterior chamber depth, AXL—axial length of the globe, BCVA—best-corrected visual acuity, MD—mean deviation (perimetry), PSD—pattern standard deviation (perimetry), RNFLT—retinal nerve fiber layer thickness, BMI—body mass index, ECC—endothelium cells count, SE—spherical equivalent.

**Table 3:** Summary of WDT results in the post-XEN and control groups.

	Post-XEN		POAG/Control		
	Mean ± SD	95% CI	Mean ± SD	95% CI	*p*-Value *
**GAT**					
Baseline	14.7 ± 2.7	13.6–15.8	15.1 ± 3.4	13.4–16.7	0.65
After 15 min	15.8 ± 2.9	14.7–16.9	16.5 ± 3.8	14.7–18.4	0.51
After 30 min	15.9 ± 3.2	14.6–17.1	16.8 ± 3.9	14.9–18.7	0.36
After 45 min	15.4 ± 2.9	14.2–16.6	16.2 ± 4.5	14.0–18.4	0.54
After 60 min	14.9 ± 2.7	13.9–15.9	16.3 ± 5.1	13.8–18.7	0.28
(end-pressure)					
**IOP_CC_**					
Baseline	15.3 ± 3.9	13.7–16.8	15.9 ± 4.6	13.7–18.2	0.63
After 15 min	17.5 ± 4.1	15.9–19.1	18.1 ± 4.2	16.0–20.1	0.57
After 30 min	17.7 ± 4.2	16.1–19.4	18.3 ± 3.9	16.4–20.2	0.62
After 45 min	16.5 ± 3.6	15.1–17.9	18.4 ± 4.8	16.0–20.7	0.06
After 60 min	16.3 ± 3.5	14.9–17.7	18.7 ± 5.8	15.8–21.5	0.11
**IOP_G_**					
Baseline	13.4 ± 4.3	11.7–15.1	13.9 ± 4.7	11.6–16.2	0.68
After 15 min	15.4 ± 4.4	13.6–17.1	15.9 ± 4.2	13.9–17.9	0.57
After 30 min	15.5 ± 4.2	13.8–17.1	16.1 ± 4.0	14.1–18.0	0.46
After 45 min	14.2 ± 3.8	12.7–15.7	15.9 ± 4.9	13.4–18.4	0.22
After 60 min	13.9 ± 3.7	12.5–15.4	16.3 ± 5.8	13.5–19.1	0.11
**CH**					
Baseline	9.4 ± 1.8	8.7–10.1	8.8 ± 1.9	7.9–9.8	0.66
After 15 min	8.9 ± 1.7	8.3–9.6	9.2 ± 1.9	8.3–10.2	0.77
After 30 min	8.8 ± 1.7	8.1–9.4	9.1 ± 1.7	8.4–9.9	0.92
After 45 min	8.9 ± 1.8	8.2–9.6	8.9 ± 2.1	7.8–9.9	0.51
After 60 min	8.9 ± 1.7	8.3–9.6	9.1 ± 2.1	8.1–10.1	0.4
**CRF**					
Baseline	8.9 ± 2.1	8.1–9.8	9.1 ± 1.7	8.3–9.9	0.87
After 15 min	9.1 ± 2.0	8.3–9.9	8.9 ± 1.8	7.9–9.7	0.96
After 30 min	9.0 ± 1.8	8.3–9.7	8.7 ± 1.5	8.0–9.4	0.6
After 45 min	8.7 ± 1.8	7.9–9.5	8.5 ± 1.8	7.6–9.4	0.99
After 60 min	8.7 ± 1.9	7.9–9.4	8.6 ± 1.9	7.7–9.5	0.74

* Mann–Whitney *U* test; GAT—Goldmann applanation tonometry, IOP_CC_—corneal-compensated intraocular pressure, IOP_G_—Goldmann-correlated, CH—corneal hysteresis, CRF—corneal resistance factor.

**Table 4:** Summary of recent studies on water drinking test after different surgical vs. medical treatment.

Source	Number of Patients	Procedure Type	GAT Baseline (mm Hg)	GAT Fluctuations (mm Hg)	GAT Amplitude (mm Hg)	GAT Range during WDT (mm Hg)	GAT End-Pressure Difference (mm Hg)	GAT Maximum (mm Hg)
Post-trabeculectomy water drinking test performance								
Danesh-Mayer et al., 2008 [18]	*N* = 30	Post-trabeculectomy	10.4 ± 2.3	Not shown	Not shown	2.2 ± 1.3	Not shown	11.7 ± 2.6
Razeghinejad et al., 2017 [16]	*N* = 30	Post-trabeculectomy	14.8 ± 2.9	4.0 ± 4.3	4.0 ± 4.3	Not shown	3.2 ± 4.7	18.8 ± 4.7
Martinez et al., 2017 [17]	*N* = 20	Post-trabeculectomy	12.3 ± 4.3	3.95 ± 2.2	3.95 ± 2.2	2.8 ± 1.6	Not shown	16.3 ± 5.6
Razeghinejad et al., 2018 [19]	*N* = 53	Post-trabeculectomy	14.1 ± 3.2	5.5 ± 4.4	Not shown	Not shown	3.1 ± 4.3	19.6 ± 6.0
Post-tube water drinking test performance								
Razeghinejad et al., 2017 [16]	*N* = 30	Post-tube	14.2 ± 3.9	5.6 ± 3.6	5.6 ± 3.6	Not shown	5.6 ± 3.6	19.7 ± 6.0
Martinez et al., 2017 [17]	*N* = 20	Post-tube	12.6 ± 4.2	3.6 ± 2.2	3.6 ± 2.2	2.8 ± 1.5	Not shown	16.2 ± 5.4
Razeghinejad et al. [19]	*N* = 31	Post-tube	14.3 ± 3.9	6.8 ± 3.4	Not shown	Not shown	5.6 ± 3.6	21.1 ± 5.8
Post-laser procedures water drinking test performance								
Kerr et al., 2016 [20]	*N* = 20	Post-SLT	14.2 ± 2.3	Not shown	Not shown	Not shown	Not shown	16.5 ± 3.2
Medical treatment water drinking test performance								
Danesh-Mayer et al., 2008 [18]	*N* = 30	Medical treatment	11.1 ± 1.8	Not shown	Not shown	5.6 ± 1.9	Not shown	17.3 ± 2.7
Razeghinejad et al., 2018 [19]	*N* = 37	Medical treatment	14.9 ± 3.1	6.9 ± 4.0	Not shown	Not shown	4.9 ± 4.6	21.8 ± 5.3
Kerr et al., 2016 [20]	*N* = 20	Medical treatment	16.9 ± 2.4	Not shown	Not shown	Not shown	Not shown	21.4 ± 3.4
Our study	*N* = 22	Medical treatment	15.1 ± 3.4	3.0 ± 2.8	3.6 ± 2.5	3.0 ± 1.9	0.8 ± 3.1	18.3 ± 4.6
Post-XEN GelStent water drinking test performance								
Our study	*N* = 27	Post-XEN GelStent	14.7 ± 2.7	2.1 ± 1.5	2.9 ± 1.3	2.4 ± 1.1	0.3 ± 1.8	16.8 ± 2.9

## Data Availability

The data will be available via online access according to the Clinical Trials registration statement.

## Supplementary Materials

The following supporting information can be downloaded at: https://www.mdpi.com/article/10.3390/jcm11010175/s1, Figure S1: Example of ORA measurement with main descriptors and parameters (solid line—applanation, dashed line—air preassure); Figure S2 Comparison of the categorized GAT peak between the post-XEN and control groups (percentage), Fisher’s exact test. Figure S3 Changes in the median of intraocular pressure measured by Goldmann applanation tonometry (GAT) in the examined groups during the water-drinking test. * *p* < 0.05, Friedmann test. Table S1. Number of participants during screening, enrolment and observation periods; Table S2. Reasons for exclusion during each period of the study by group.

## Clinical Trial Registration

ClinicalTrials.gov Identifier: NCT03904381.
