# Supplementary material for: Short-Time Changes of Intraocular Pressure and Biomechanics of the Anterior Segment of the Eye during Water Drinking Test in Patients with XEN GelStent
Source: J Clin Med. 2021 Dec 29;11(1):175. doi: 10.3390/jcm11010175 (PMC8745870; doi:10.3390/jcm11010175)
Supplement: Supplementary file 1 [file jcm-11-00175-s001.zip › jcm-1482899-supplementary.pdf]

Supplementary Materials

Table S1. Number of participants during screening, enrolment and observation periods.

	Clinical data base*	Screening period	Enrolled	12 months of follow-up period
Post-XEN	50	39	27	27
M-POAG	86	51	22	22

M-POAG – primary open angle glaucoma matched with post-XEN patient in the terms of age, sex, refractive error, axial length, glaucoma progression, and retinal nerve fibre layer thickness; *during Clinical data base phase patients were selected based on their historical data, that were checked in the screening period according to the inclusion and exclusion criteria.

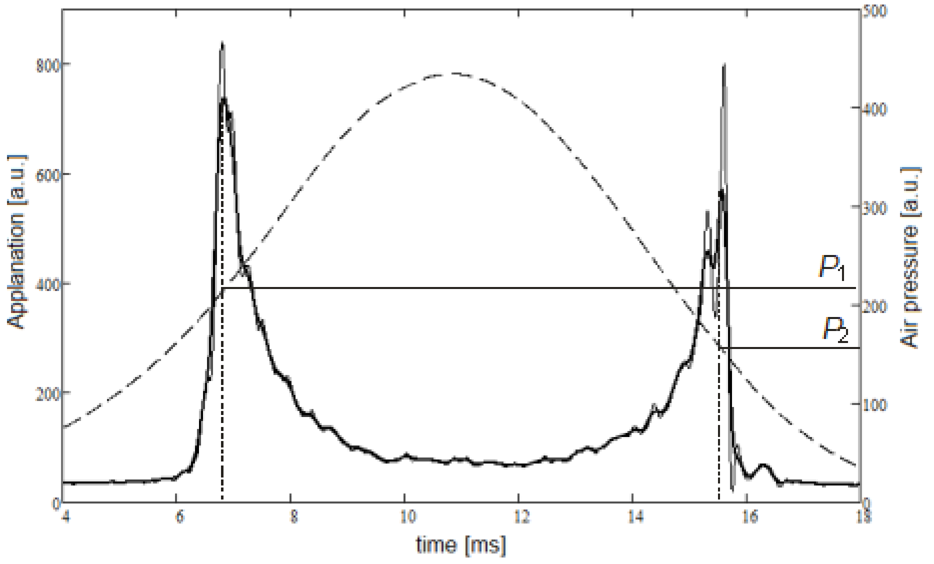

Figure S1. Example of ORA measurement with main descriptors and parameters (solid line – applanation, dashed line – air pressure).

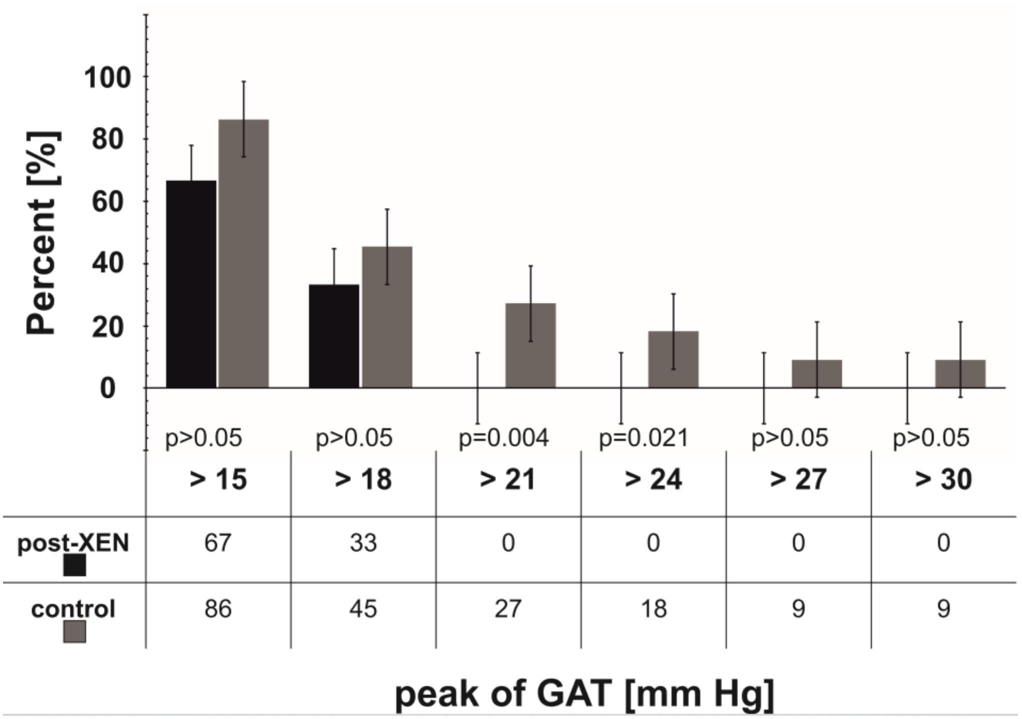

Figure S2. Comparison of the categorized GAT peak between the post-XEN and control groups (percentage), Fisher's exact test.

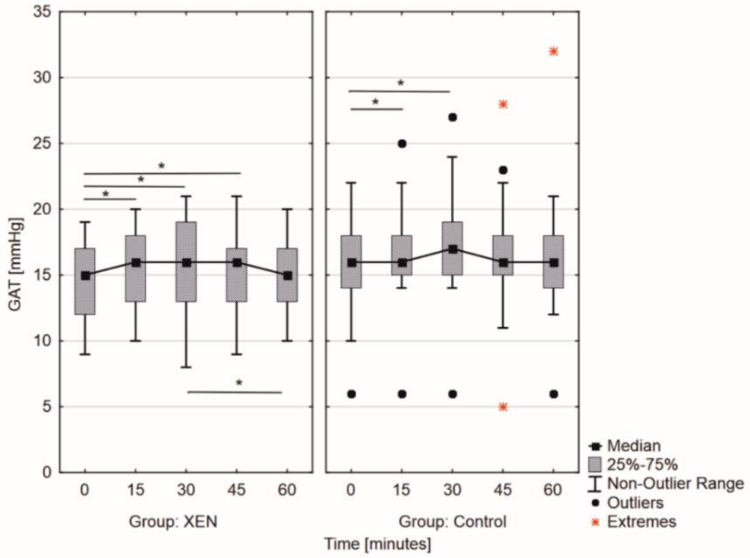

Figure S3. Changes in the median of intraocular pressure measured by Goldmann applanation tonometry (GAT) in the examined groups during the water-drinking test. *p<0.05, Friedmann test.

Table S2 Reasons for exclusion during each period of the study by group.

		Reasons for exclusion							
		Inability to contact with patient	Lack of consent	Non-compliance	Not matched with post-XEN	Reduction of IOP compared to the pre-treatment**	Progression within last 3 months	Change in medication within last 3 months	Systemic medication within last 3 months
Clinical data base*	Post-XEN	6	4	1	-	-	-	-	-
	M-POAG	22	9	4	-	-	-	-	-
Screening period	Post-XEN	0	0	1	-	2	0	5	4
	M-POAG	0	0	1	14	5	1	4	4
Enrolment period	Post-XEN	0	0	0	-	0	0	0	0
	M-POAG	0	0	0	-	0	0	0	0
12 months follow-up	Post-XEN	0	0	0	-	0	0	0	0
	M-POAG	0	0	0	-	0	0	0	0

M-POAG – primary open angle glaucoma matched with post-XEN patient in the terms of age, sex, refractive error, axial length, glaucoma progression, and retinal nerve fiber layer thickness; *during Clinical data base patients were selected based on their historical data, that were checked in the screening period according to the inclusion and exclusion criteria. ** reduction of IOP compared to the pre-treatment measurements of at least ≥20% baseline IOP and ≤21 mmHg.

Description 1 – parameters that can influence corneal hysteresis

The ocular response analyzer (ORA; Reichert Ophthalmic Instruments, Inc., Buffalo, NY, USA), a non-contact tonometer that enables measurement of intraocular pressures (IOP_G and IOP_{cc}), and two biomechanical parameters, namely corneal hysteresis (CH), which indicates corneal viscoelasticity, and corneal resistance factor (CRF), which is related to corneal elasticity. These four parameters are calculated from two applanation pressures ("inward" P1 and "outward" P2) obtained during corneal deformation (recorded within 25 ms) resulting from air jet pulse. The average of received pressures (P1 and P2) is the Goldmann-correlated IOP (IOP_G). One of the obtained measurements that shows no diurnal variation is CH that along with mathematically estimated CRF is considered responsible for viscoelastic properties of the cornea (viscosity and elasticity, respectively).

Several factors have been described previously as important for corneal hysteresis measurements and analysis. Corneal shape alongside with corneal thickness are the most important factors [1–3]. Wong et al. in 2011 describe influence of corneal astigmatism, corneal curvature and meridional difference on the corneal biomechanics. In this work a head rotation has been introduced to obtain the comparable results. Each rotation whether it was by 10, 20 or 30-degree were significant for corneal hysteresis and corneal resistance factor measurements[1]. Rosa et al. in 2015 evaluated the influence of central corneal thickness (CCT) and keratometry readings (KM) on CH and CRF. According to this results the strongest influence showed KM ($r=0.292$ and $r=0.248$, $p<0.001$ and $p=0.002$ respectively for CH and CRF) compared to for CCT ($r=0.016$ and $r=0.022$, $p<0.001$ respectively for CH and CRF) [2]. The corneal structure (such as corneal matrix, cellular density of keratocytes and endothelial cells) was considered by some authors as another significant factor that can influence CH and CRF. Especially, this seems to be important as one of the function of endothelial cell is to prevent corneal oedema to maintain corneal transparency [4]. However in this respect there are no define results [3, 5].

Age-dependent changes in ORA measurements have also been evaluated. Moreover, the decrease in CH and CRF values have been observed with advancing age[6–8]. The statistically significant decrease in CH has been estimated at < 0.1 mmHg/year[8]. Kotecha et al. (2006) introduced a completely IOP-independent constant corneal factor (CCF) that exhibited an age-dependent decrease at the level of < 0.3 mmHg/decade (< 0.03 mmHg/year). Experimental *ex vivo* studies have shown an age-related change in the corneal collagen fibril biomechanics that may contribute to an increased stiffness of the cornea with age[9, 10]. *In vivo* endothelial specular microscopic studies have demonstrated corneal signs that indicate an increased corneal stiffness with age[11, 12].

The impact of systemic disease on measurements of biomechanical parameters and IOP has been described in previous studies. In most studies, patients with diabetes mellitus (DM) exhibited an increase in CH, CRF, CCT, and IOP (IOP_{CC}, IOP_G, and GAT)[6, 13–18]. Moreover, studies reported a decrease in CH[14, 15]. Another study reported a weak but statistically significant correlation between CH, CRF, and non-fasting serum glucose[18]. On the contrary, more recent studies have reported no statistical difference in the biomechanical properties between the DM group and matched healthy controls[15, 19]. Some authors indicated that poor glucose control in DM affected corneal biomechanics[20, 21]. Similar findings were presented when HbA1c was considered[22]. The reported argumentation could be the cause for differences in the reported study groups. Another study described the impact of systemic scleroderma, systemic lupus erythematosus, secondary Sjögren's syndrome, Hashimoto's thyroiditis, or Marfan syndrome on the biomechanical properties of the cornea[23].

Due to the widely described in present paragraph factors that possibly influence biomechanical measurements, in our study the preselection of included patients was fierce. Nearly 54% and only 26% respectively in the post-XEN and in the control groups included in the clinical data base phase were finally enrolled.

Supplementary bibliography:

1. Wong Y, Lam AKC (2011) Influence of corneal astigmatism, corneal curvature and meridional differences on corneal hysteresis and corneal resistance factor. Clin Exp Optom 94:418–424. https://doi.org/10.1111/j.1444-0938.2011.00591.x
2. Rosa N, Lanza M, De Bernardo M, et al (2015) Relationship Between Corneal Hysteresis and Corneal Resistance Factor with Other Ocular Parameters. Semin Ophthalmol 30:335–339. https://doi.org/10.3109/08820538.2013.874479
3. Gatzioufas Z, Labiris G, Stachs O, et al (2013) Biomechanical profile of the cornea in primary congenital glaucoma. Acta Ophthalmol 91:e29-34. https://doi.org/10.1111/j.1755-3768.2012.02519.x
4. Mergler S, Pleyer U (2007) The human corneal endothelium: new insights into electrophysiology and ion channels. Prog Retin Eye Res 26:359–378. https://doi.org/10.1016/j.preteyeres.2007.02.001
5. Hurmeric V, Sahin A, Ozge G, Bayer A (2010) The relationship between corneal biomechanical properties and confocal microscopy findings in normal and keratoconic eyes. Cornea 29:641–649. https://doi.org/10.1097/ICO.0b013e3181c11dc6
6. Narayanaswamy A, Chung RS, Wu R-Y, et al (2011) Determinants of corneal biomechanical properties in an adult Chinese population. Ophthalmology 118:1253–1259. https://doi.org/10.1016/j.ophtha.2010.12.001
7. Kamiya K, Shimizu K, Ohmoto F (2009) Effect of aging on corneal biomechanical parameters using the ocular response analyzer. J Refract Surg 25:888–893. https://doi.org/10.3928/1081597X-20090917-10
8. Hager A, Wegscheider K, Wiegand W (2009) Changes of extracellular matrix of the cornea in diabetes mellitus. Graefes Arch Clin Exp Ophthalmol 247:1369–1374. https://doi.org/10.1007/s00417-009-1088-4
9. Daxer A, Misof K, Grabner B, et al (1998) Collagen fibrils in the human corneal stroma: structure and aging. Invest Ophthalmol Vis Sci 39:644–648
10. Malik NS, Moss SJ, Ahmed N, et al (1992) Ageing of the human corneal stroma: structural and biochemical changes. Biochim Biophys Acta 1138:222–228. https://doi.org/10.1016/0925-4439(92)90041-k
11. Sherrard ES, Novakovic P, Speedwell L (1987) Age-related changes of the corneal endothelium and stroma as seen in vivo by specular microscopy. Eye (Lond) 1 (Pt 2):197–203. https://doi.org/10.1038/eye.1987.37
12. Elsheikh A, Wang D, Brown M, et al (2007) Assessment of corneal biomechanical properties and their variation with age. Curr Eye Res 32:11–19. https://doi.org/10.1080/02713680601077145
13. Castro DPE, Prata TS, Lima VC, et al (2010) Corneal viscoelasticity differences between diabetic and nondiabetic glaucomatous patients. J Glaucoma 19:341–343. https://doi.org/10.1097/IJG.0b013e3181b4caa1
14. Sahin A, Bayer A, Ozge G, Mumcuoğlu T (2009) Corneal biomechanical changes in diabetes mellitus and their influence on intraocular pressure measurements. Invest Ophthalmol Vis Sci 50:4597–4604. https://doi.org/10.1167/iovs.08-2763
15. Bekmez S, Kocaturk T (2018) Higher Intraocular Pressure Levels Associated With Lower Hysteresis In Type 2 Diabetes. Open Ophthalmol J 12:29–33. https://doi.org/10.2174/1874364101812010029
16. Ramm L, Herber R, Spoerl E, et al (2019) Measurement of Corneal Biomechanical Properties in Diabetes Mellitus Using the Ocular Response Analyzer and the Corvis ST. Cornea 38:595–599. https://doi.org/10.1097/ICO.0000000000001879
17. Goldich Y, Barkana Y, Gerber Y, et al (2009) Effect of diabetes mellitus on biomechanical parameters of the cornea. J Cataract Refract Surg 35:715–719. https://doi.org/10.1016/j.jcrs.2008.12.013
18. Kotecha A, Oddone F, Sinapis C, et al (2010) Corneal biomechanical characteristics in patients with diabetes mellitus. J Cataract Refract Surg 36:1822–1828. https://doi.org/10.1016/j.jcrs.2010.08.027
19. Beato JN, Esteves-Leandro J, Reis D, et al (2019) Structural and Biomechanical Corneal Differences between Type 2 Diabetic and Nondiabetic Patients. J Ophthalmol 2019:3764878. https://doi.org/10.1155/2019/3764878
20. Pérez-Rico C, Gutiérrez-Ortíz C, González-Mesa A, et al (2015) Effect of diabetes mellitus on Corvis ST measurement process. Acta Ophthalmol 93:e193-198. https://doi.org/10.1111/aos.12530
21. Scheler A, Spoerl E, Boehm AG (2012) Effect of diabetes mellitus on corneal biomechanics and measurement of intraocular pressure. Acta Ophthalmol 90:e447-451. https://doi.org/10.1111/j.1755-3768.2012.02437.x
22. Yazgan S, Celik U, Kaldırım H, et al (2014) Evaluation of the relationship between corneal biomechanic and HbA1C levels in type 2 diabetes patients. Clin Ophthalmol 8:1549–1553. https://doi.org/10.2147/OPTH.S67984
23. Emre S, Kayikçioğlu O, Ateş H, et al (2010) Corneal hysteresis, corneal resistance factor, and intraocular pressure measurement in patients with scleroderma using the reichert ocular response analyzer. Cornea 29:628–631. https://doi.org/10.1097/ICO.0b013e3181c3306a.
